# Relationship between the Ionization Degree and the Inter-Polymeric Aggregation of the Poly(maleic acid-*alt*-octadecene) Salts Regarding Time

**DOI:** 10.3390/polym12051036

**Published:** 2020-05-02

**Authors:** Isabella Reyes, Maria M. Palacio, Cristhian J. Yarce, Jose Oñate-Garzón, Constain H. Salamanca

**Affiliations:** 1 Laboratorio de Diseño y Formulación de Productos Químicos y Derivados, Departamento de Ciencias Farmacéuticas, Facultad de Ciencias Naturales, Universidad ICESI, Calle 18 No. 122-135, Cali 760035, Colombia; isabellarm@hotmail.es (I.R.); palacio-198@hotmail.com (M.M.P.); cjyarce@icesi.edu.co (C.J.Y.); 2 Grupo de Investigación en Química y Biotecnología (QUIBIO), Facultad de Ciencias Básicas, Universidad Santiago de Cali, calle 5 No. 62-00, Cali 760035, Colombia; jose.onate00@usc.edu.co

**Keywords:** amphiphilic polymeric material, dynamics of polyelectrolyte, inter-polymeric aggregation, ionization degree, poly(maleic acid-*alt*-octadecene) salts

## Abstract

Alternating amphiphilic copolymers are macromolecular systems with a polarity duality in their structure, since they are generally formed by alternating segments corresponding to a potential electrolyte group and an alkyl (aliphatic or aromatic) group. These systems, depending on the ionization degree, as well as the time, may form different types of intra and interpolymeric aggregates in aqueous media. Therefore, this study, which in fact is the continuation of a previously reported work, is focused on establishing how the ionization degree of the sodium and potassium salts of the poly(maleic acid-*alt*-octadecene) affect zeta potential, pH, electrical conductivity, particle size, polydispersity index, and surface tension over time. The results showed that polymeric salts with a high ionization degree in aqueous media formed homogeneous systems with bimodal sizes and high zeta potential values, which tended to quickly become less negative, lowering the pH and slightly increasing the electrical conductivity; while systems with low ionization degree lead to the opposite, forming heterodispersed systems with several populations of particle sizes, high polydispersity, low zeta potential values, neutral and invariable pH values, and high electrical conductivity values. Consequently, these results suggest that the values of particle size, polydispersity index, zeta potential, pH, and electrical conductivity change regarding the polymeric ionization degree, as well as the time. Therefore, such variables should be considered and controlled when working with this kind of polymeric materials.

## 1. Introduction

Amphiphilic polymeric materials are macromolecular systems that describe a polarity duality regarding to the distribution of several polar and non-polar functional groups in their polymer chains [1,2]. Depending on such distribution, it is possible to obtain different types of polymeric systems, such as block copolymers [3], hydrophobically modified polymers (HMPs) [4,5,6,7,8], and alternating amphiphilic copolymers [9,10]. In the case of block copolymers, they are formed by a segment of polar repeating units (neutral or ionizable) and another segment of non-polar repeating units. In contrast, hydrophobically modified polymers (HMPs) can be homopolymers or copolymers that are initially hydrophilic, but subsequently are transformed by the incorporation of a hydrophobic component in their polymeric chain. These modifications are usually carried out by esterification or amidation reactions with aliphatic alcohols and amines, respectively [11]. Finally, there are the alternating amphiphilic copolymers which are the central focus of this study. These polymers are formed by an alternating distribution of a polar segment that can be neutral or ionizable and a non-polar segment that can be aliphatic or aromatic. These polymeric materials describe different behaviors regarding to the intra and inter-polymer aggregation in aqueous medium. For instance, block copolymers tend to form self-assembling structures highly organized (polymeric micelles) [12,13,14], while PMHs and alternating amphiphilic copolymers tend to form multiple intramolecular as well as intermolecular aggregates [15,16,17]. These aggregates can be formed depending on several intrinsic factors, such as the polymer concentration and the balance between the charge fraction and the hydrophobic character in the polymer chains. Likewise, such aggregates also depend on extrinsic factors, such as pH, ionic strength, and temperature. In the case of the polymeric materials derived from the poly(maleic acid-*alt*-octadecene) salts, it has been reported that when these polymers have high ionization degree, being in aqueous dilution, they can form hydrophobic pseudo-phases [10,18]. Such pseudo-phases have been described mainly by indirect methodologies, such as fluorescent probes [19,20] and the partitioning of organic substrates between the aqueous medium and such polymeric pseudo-phases [18,21]. However, it is not entirely clear if these polymers tend to form mainly intramolecular or intermolecular aggregates or a mixture of both. Similarly, the intrinsic and extrinsic conditions that lead to the formation of one or another type of aggregates have not yet been fully described. Hence, understanding this type of system remains a challenging problem and although several studies has been reported, there are very few studies that describe in an explicitly and parameterized way, how such polymeric materials with different ionization degrees may affect several physicochemical properties regarding time. Accordingly, this study, which at first impression seems classic and without novelty, in fact is a work that complements a previous research, where a relationship between the polymeric ionization degree and powder and surface properties in materials derived from poly(maleic anhydride-*alt*-octadecene) was established [22]. Therefore, this work provides a detailed parameterization about the changes that take place in several physicochemical properties (particle size, polydispersity, zeta potential, pH, electrical conductivity, and surface tension) for the sodium and potassium salts of poly(maleic acid-*alt*-octadecene) regarding the polymeric ionization degree, the type of counterion, and the time.

## 2. Material and Methods

### 2.1. Materials

The sodium and potassium salts of poly(maleic acid-*alt*-octadecene) with different ionization degrees (ID) and named like PAM-18Na (monomeric unit: 412 g/mol, *M*_w_ 30–50 kD) and PAM-18K (monomeric unit: 428 g/mol, *M*_w_ 30–50 kD), were supplied by the laboratory of Design and Formulation of Chemical Products from Icesi University (Cali, Colombia), and used as received. Likewise, the synthesis, chemical characterization, as well as the determination of the ionization degree of these polymeric materials were reported in a previous work [22]. In the case of PAM-18Na, the ID were 22%, 63%, and 95%; whilst the PAM-18K, the ID were 20%, 52%, and 99%. On the other hand, polymer dispersions were made using type I water, obtained from a purification system (Arium pro-Sartorius Stedim biotechnology VF, Göttingen, Germany).

### 2.2. Turbidimetric Solubility Test

Turbidimetric solubility [23,24] for PAM-18Na and PAM-18K with different ionization degrees were determined in triplicate, using a UV–Vis spectrophotometer coupled to a thermostat (Shimadzu 1800, Shimadzu Corporation, Kyoto, Japan). For this, the polymeric salts were independently dispersed in ultra-pure water at different concentrations depending on the ionization degrees. Low ID polyelectrolytes were prepared between 0.01 and 20.00 mg/mL, while high ID polyelectrolytes were prepared between 3.00 and 130.00 mg/mL. All systems were initially assisted with a homogenizer (Gehaka^®^ D-160, São Paulo, Brazil) at 4000 rpm for 10 min. Subsequently, each system was left on an orbital shaker (Unimax 1010, Inkubator 1000, Heidolph Instruments, Schwalbach, Germany) at 25 °C and 600 rpm for 24 h. Once the maximum agitation time was reached, the turbidimetric solubility value was immediately determined from measures of transmittance percentage (%T) at 600 nm, establishing like reference point, the value of concentration where the %T changed abruptly from 100% to a lower value. This value was determined through the evaluation of the first derivative of the %T vs polymer concentration graph. Thus, the turbidimetric solubility was taken as reference concentration for the preparation of the rest polymeric dispersions in the subsequent studies.

### 2.3. Change of Polyectrolyte Physicochemical Variables over Time

All measurements were made in triplicate with freshly prepared samples, which were previously left in an orbital shaker (Unimax 1010, Heidolph Instruments, Schwalbach, Germany) at 25 °C and 600 rpm for 24 h. Likewise, each sample evaluated over time was left sealed, in the dark, at rest and at a controlled temperature of 25 ° C, before carrying out any measurement.

#### 2.3.1. Zeta Potential, Electrical Conductivity, and pH

Zeta potential measurements were carried out using a Zetasizer nano ZSP (Malvern Instruments, Worcestershire, UK) at 25 ± 2 °C, with equilibration times of 120 s in a DTS 1070 capillary cell. This technique uses Doppler laser microelectrophoresis as a principle, where an electric field is applied to a solution of molecules or a dispersion of particles, which then move with a velocity related to its zeta potential. This speed is measured using an interferometric laser technique (light scattering for phase analysis), which allows the calculation of electrophoretic mobility, used to obtain the value of the zeta. For these experiments, the attenuator position and intensity were set automatically. The samples were prepared using a polyelectrolyte amount between 0.5 and 1 mg, which was dispersed in 10 mL of ultra-pure water and manually stirred. Then, a 50 µL aliquot was taken and diluted with 1 mL of ultra-pure water before each zeta potential measurement. Conversely, the electrical conductivity and the pH were determined using a CR-30 conductivity meter and a Starter-2100 pH meter, respectively. All measurements were performed at approximately 23 ± 1 °C (room temperature).

#### 2.3.2. Particle Size and Polydispersity

Particle size and polydispersity index (PDI) were determined using a Zetasizer nano ZSP (Malvern Instrument, Worcestershire, UK) with a red helium/neon laser (633 nm), where 10 µL of each polyelectrolyte sample (with concentration between 0.5 mg/mL and 1.0 mg/mL) was dispersed in 10 mL of ultra-pure water. Besides, the particle size was measured using a scattering angle of 173° and a quartz flow cell (ZEN0023) at 25 °C, where the instrument reports the particle size as the mean particle diameter (z-average) and PDI ranging from 0 (monodisperse) to 1 (very broad distribution). All experiments were measured by dynamic light scattering (DLS), where the diffusion of Brownian moving particles was determined, and subsequently, it is converted to values of size and size distribution through the Stokes–Einstein ratio. Also, this technique measures the fluctuations in the intensity of the dispersion as a function of time, where the distribution of the observed intensity is analyzed by a correlation function. 

#### 2.3.3. Surface Tension

The optical contact angle measuring and contour analysis systems OCA15EC from Dataphysics (Software SCA22 version 4.5.14), coupled with a needle SNP 165/119 was employed for the determination of surface tension, in PAM-18Na and PAM-18K polyelectrolytes with high ID in aqueous media, through the pendant drop method [25]. Each measurement was performed at approximately 23 ± 1 °C (room temperature) and 60% ± 5% relative humidity. Likewise, the polymer solutions used in this study were recently prepared in a concentration range of 0.01 M and 0.15 M.

### 2.4. Graphs and Statistical Analysis

The determination of the average values, the standard deviations and the graphs were made using the Microsoft Excel, Microsoft Office, Office 365, and GraphPad prism 8 software. Likewise, the effects of the polyectrolyte counterion (Na+ or K+) on the apparent solubility, zeta potential, electrical conductivity, pH particle size, PDI, and the AUC values of the surface tension vs. the polyelectrolyte concentration profile for the same time and ionization degree were evaluated, using the one-way ANOVA test. 

## 3. Results and Discussion

### 3.1. Turbidimetric Solubility Test

**Figure 1:**
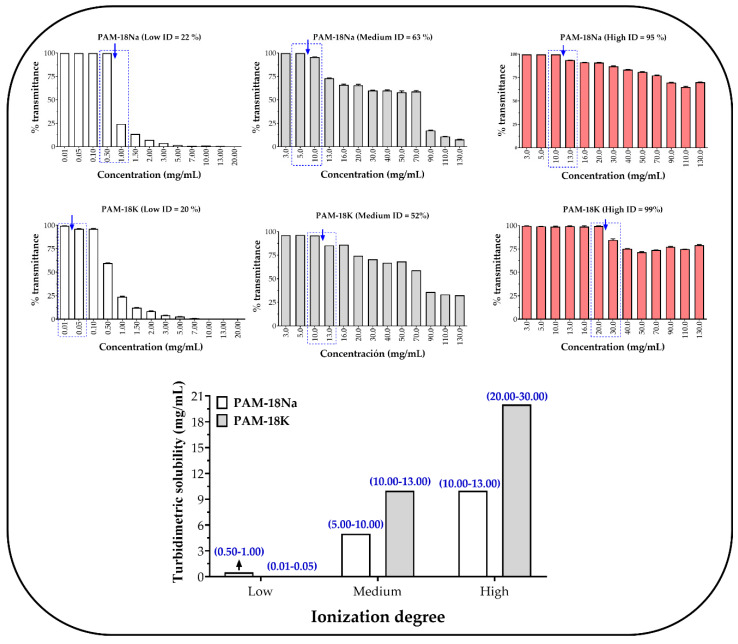
Values of turbidimetric solubility for PAM-18Na and PAM-18K polyelectrolytes with different ionization degree (ID) in aqueous media.

The results of the turbidimetric solubility, and which corresponds to an approximate or apparent value, are presented in Figure 1, where the concentration zones corresponding to the change in transmittance from ~100% to a lower value are presented, as well as their respective comparison between each polyelectrolytic system. In addition, Figure 1 shows that the increase in the polymeric ionization degree seems to increase the apparent solubility in the polyelectrolytes, being greater in those systems with the potassium counterion. These results can be explained considering that, when the polyelectrolytes have a great quantity of ionic groups, these may generate a larger amount of ion-dipole polymer–solvent interactions, favoring the solubility in aqueous medium [26,27]. In contrast, the polyelectrolytes with a low ionization degree, have a very weak polymer–solvent interactions (hydrogen bonds), where the hydrophobic effect provided by the polymeric lateral alkyl chains prevail, affecting the aqueous solubility.

Regarding to the polyelectrolyte counterion effect, it is possible to observe that the PAM-18K polymer may cause changes in %T at higher concentration than the PAM-18Na polyelectrolyte and where the apparent solubility is described as a value range. Thus, the PAM-18K polymers displayed values between 0.01 and 0.50 mg/mL (low ID), 10.00 and 13.00 mg/mL (medium ID), and 20.00 and 30 g/mL (high ID); whilst the PAM-18Na polymer showed values between 0.5 and 1.00 mg/mL (low ID), 5.00 and 10.00 mg/mL (medium ID), and 10.00 and 13 mg/mL (high ID). Such differences in the apparent solubility can be explained considering that potassium ions have a larger ionic radius and therefore, they establish weaker interactions with the anionic polyelectrolyte, forming ion pairs that can be more easily separated by the aqueous medium, favoring the solvation effect.

On the other hand, and based on the turbidimetric solubility, the concentration value for subsequent studies was established. Therefore, a polymer concentration of 1.00 mg/mL was defined for all systems, except those with a low ionization degree where a concentration of 0.50 mg/mL was defined.

### 3.2. Change of Polyectrolyte Physicochemical Variables over Time

#### 3.2.1. Zeta Potential, pH, and Electrical Conductivity Measurements

**Figure 2:**
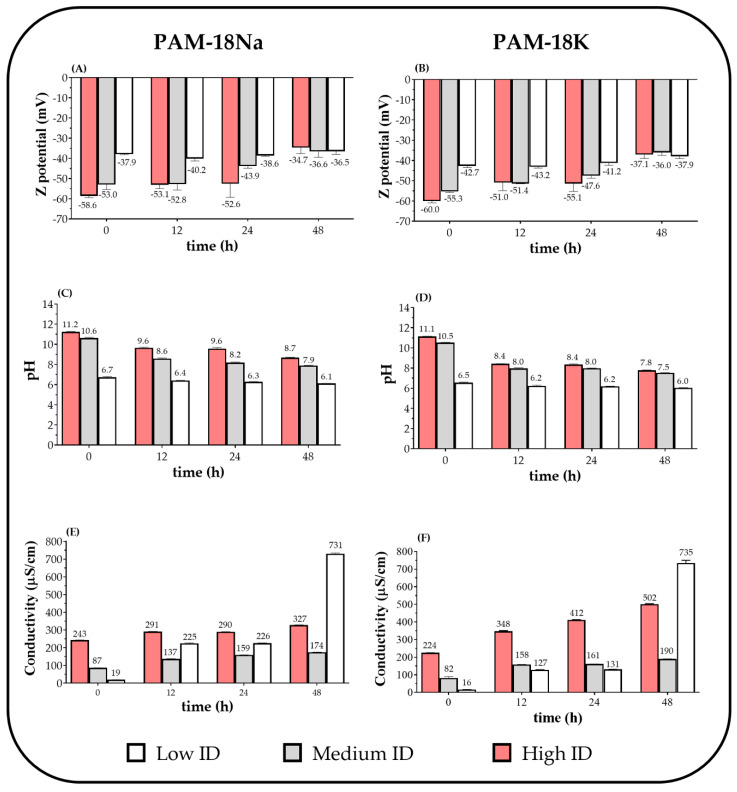
Change of: (**A**,**B**) zeta potential, (**C**,**D**) pH, and (**E**,**F**) conductivity over time for PAM-18Na and PAM-18K polyelectrolytes with different ionization degree (ID) in aqueous media.

Figure 2A,B show the zeta potential results for the anionic polyelectrolytes PAM-18Na and PAM-18K in aqueous medium regarding time. 

Zeta potential results are very interesting, since they show a relationship between the ionization degree and the zeta potential, where the increase in the ID leads to more negative values of zeta potential. Likewise, it was observed that, over time, these values changed depending on the ID and the type of polyelectrolyte. For instance, the PAM-18Na systems with high ID, the zeta potential changed from ~−59 to ~−35 mV, while with a medium ID change from ~−53 to ~−37 mV and with a low ID, it remained practically unchanged at ~−38 mV. Similarly, it was observed that PAM-18K systems with high ID, the zeta potential changed from ~−60 to ~−37 mV, while with a medium ID change from ~−55 to ~−36 mV and with a low ID slightly changed from ~−43 to ~−38 mV. Thus, in polymer systems with high ID, the polymeric interface became more depolarized over time. These zeta potential results are very consistent with those obtained regarding to pH (Figure 2C,D), where it was found that in both polyelectrolytes with high and medium ionization degrees, the aqueous media pH also decrease, going from ~11.2 to ~8.0 and from ~10.5 to ~7.5, respectively. Whereas with the low ionization polyelectrolytes, the pH of the medium changed slightly from ~6.7 to ~6.0. 

On the other hand, the results of the electrical conductivity for the PAM-18Na and PAM-18K polyelectrolytes in aqueous medium regarding to time are shown in Figure 2E,F, where a contrary behavior is described to those observed in the zeta potential and pH. In this case, it was observed that the electrical conductivity increased over time, being higher in those polyelectrolytes with lower ionization degrees. Thus, at zero time, the electrical conductivity described very similar values in both polyelectrolytes around 230 µS/cm (high ID), 85 µS/cm (medium ID), and 18 µS/cm (low ID). However, over the time, the systems with low ionization degree showed a considerable increase in conductivity, reaching values of ~730 µS/cm in both polyelectrolytes. While in the rest of the polyelectrolytes (high and medium ID) showed a moderate increase in conductivity going from ~327 µS/cm (PAM-18Na) to ~502 µS/cm (PAM-18K) for polyelectrolytes with high ID and from ~174 µS/cm (PAM-18Na) to ~190 µS/cm (PAM-18K) for polyelectrolytes with medium ID. 

**Figure 3:**
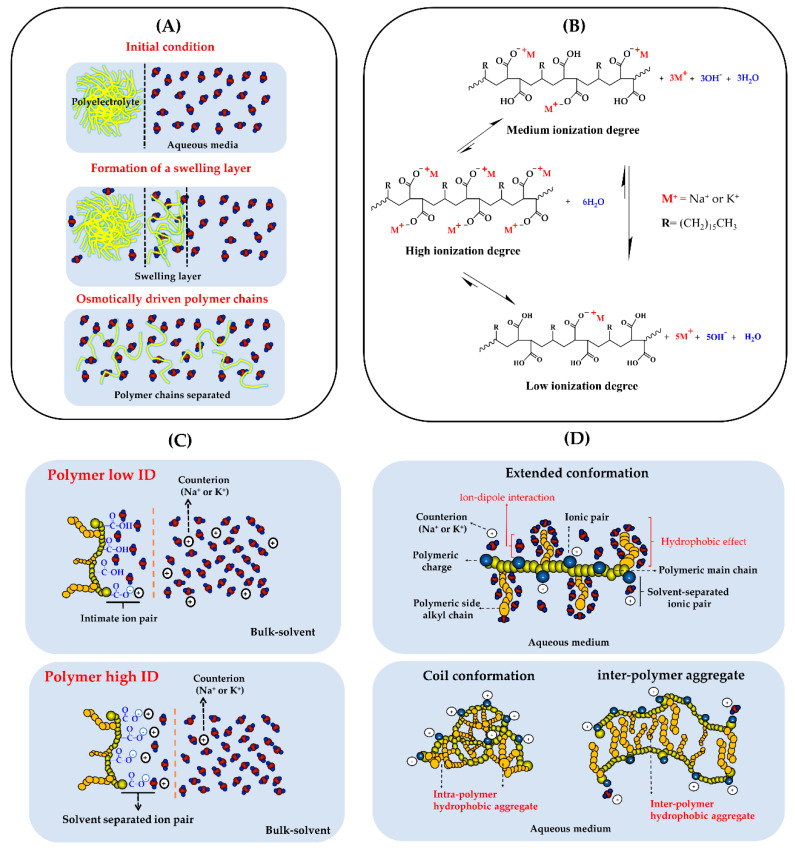
Scheme of the processes of: (**A**) Polyelectrolyte solubilization; (**B**) Thermodynamic equilibrium between carboxylate and carboxylic acids groups of polyelectrolytes; (**C**) Migration of the counterions from polyelectrolyte interface towards the bulk-solvent; (**D**) Configurations of polyelectrolytes solubilized in aqueous medium.

All these results of zeta potential, pH, and electrical conductivity can be explained considering a series of situations that occur between the polyelectrolyte and the aqueous medium (Figure 3A). At zero time, when polyelectrolytes are added to the aqueous media, a first thermodynamic condition is promoted, where a slight separation of the polymeric counterions happens due to the effect of the solvent (solvent-separated ion pairs) [28]. Subsequently, the aqueous solvent penetrates the polymeric interface, leading to rapid swelling of the polyelectrolytic chains forming a partial solvation [29,30]. Then, the polymer chains are osmotically driven to the bulk-solvent and where several types of interactions take place between the polymer backbone (carboxylate groups) and the side chains (allylic groups) and the aqueous medium. [31,32,33]. Therefore, the aqueous solubilization is generated by a balance between the attractive interactions (ion-dipole and hydrogen bonding) provided by the polar groups and the hydrophobic effect given by the alkyl side chains [34]. Once the maximum solubilization condition is reached, multiple thermodynamic equilibria are generated between the carboxylate groups of the PAM-18Na and PAM-18K polyelectrolytes and the aqueous medium. Hence, the carboxylate groups are neutralized by taking protons from the media, generating hydroxyl ions that take other protons from the polymeric carboxylic acids until reaching a state of multiple thermodynamic equilibria, as depicted in Figure 3B. Thus, depending on the initial ionization degree, PAM-18Na and PAM-18K materials may be considered as strong, relatively strong and weak polyectrolyte. In this way, these polymers with high ID have a greater amount of carboxylate group (COO^−^) than carboxylic acid group (COOH) and therefore, they can be considered as a strong polyelectrolyte. While polymers with intermediate ID, the ratio between COO^−^ and COOH groups is closer and such systems can be considered as relatively strong polyelectrolyte. In contrast, polymers with low ID have a greater amount of COOH than COO^−^ and consequently, they can be considered as weak polyelectrolyte. However, over time, all these polyelectrolytes change their initial thermodynamic equilibria, where the polyelectrolytes that acted as strong (high ID) and relatively strong (medium ID) are transformed into weak polyelectrolytes (low ID). Likewise, the anionic polyelectrolytes PAM-18Na and PAM-18K may generate polarized interfaces, where the counterions (Na^+^, K^+^) are adhered, forming layers of intimate ion pairs that affect their mobility in the aqueous medium [35,36]. Thus, polyelectrolytes with high ionization degrees acquire more charge in their structure, which produces a strong adhesion of the counter ions in the polyelectrolyte, affecting its mobility towards the ion zone in solution, reducing electrical conductivity. In contrast, polyelectrolytes with low ionization degrees have a low amount of carboxylate groups that produce a lower charge in their structure, facilitating the migration of ions into the solution, and increasing electrical conductivity (Figure 3C). 

Consequently, when the polyelectrolyte is completely solubilized in the aqueous medium, several types of polymeric conformations can be achieved depending on the distribution of charges in the polymeric backbone. For instance, when the polyelectrolyte has many charges close to each other, it extends in order to reduce such electrostatic repulsion. On the contrary, when the charges in the polyelectrolyte is low, the hydrophobic effects between the side chains and the aqueous medium prevail, leading to aggregation, which can be intra and interpolymeric (Figure 3D). Finally, it is important to mention that the measurements of zeta potential, pH and conductivity for ultra-pure water were also carried out as a control. However, no significant changes were observed. Therefore, the changes of such variables were exclusively attributed to the respective PAM-18Na and PAM-18K polymeric systems.

#### 3.2.2. Particle Size and Polydispersity

**Figure 4:**
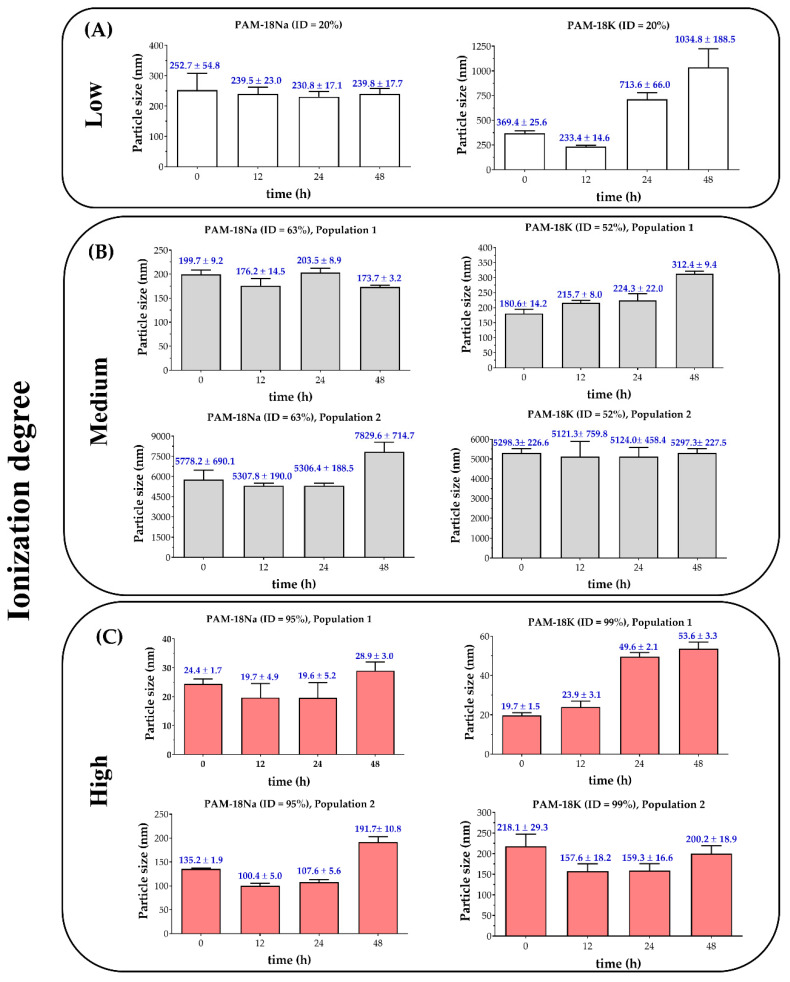
Particle size for PAM-18Na and PAM-18K polyelectrolytes with different ionization degree (ID) in aqueous media. (**A**) Low ionization degree. (**B**) Medium ionization degree. (**C**) High ionization degree.

The particle size results for the PAM-18Na and PAM18K polyelectrolytes in aqueous medium are shown in Figure 4, where it was found that the ionization degree, the type of polyelectrolyte and the time significantly affect this property. In the case of the PAM-18Na and PAM-18K polyelectrolytes with low ID (Figure 4A), highly polydisperse systems (PDI ~1) were obtained with different populations of particle size, which were corroborated by intensity, as well as by volume distribution. The results showed that most of these particle size populations were greater than 10 µm (not determinable by the instrument), and only one population could be measured, exhibiting sizes smaller than 400 nm with a volume distribution <30%. In addition, it was observed that, over time, such populations remained between ~240 and ~253 nm for the PAM-18Na, while for PAM-18K, such population sizes increased, going from ~369 to ~1035 nm. In contrast, systems with medium and high ionization degrees described bimodal size populations with fluctuating polydispersities, but always greater than 0.3 and where smaller populations had the largest volume distribution (>60%).

In the case of the PAM-18Na polyelectrolytes with medium ionization degree (Figure 4B), it was found that the smallest populations were ~200 nm, while the largest populations were between ~5000 and ~8000 nm. Furthermore, it was found that over time, the smaller population fluctuated slightly between ~174 and ~204 nm, while the larger population increased, going from ~5306 to ~7829 nm. Regarding the PAM-18K polyelectrolyte, these also described a similar behavior, where the smallest population increased slightly from ~180 to ~312 nm, while the largest population remained practically constant between ~5100 and ~5300 nm. 

Regarding the PAM-18Na polyelectrolyte with high ionization degree (Figure 4C), two populations were observed. The first one corresponding to a very small population with a particle size between ~20 and ~30 nm, and the largest population between ~100 and ~200 nm. Furthermore, it was found that, over time, the smallest population remained almost unchanged, while the largest population increased from ~135 to ~192 nm. Similarly, the PAM-18K polyelectrolyte also showed a population with a very small particle size, which increased over time, going from ~20 to ~54nm, while the second population remained fluctuating between ~158 and ~218 nm.

On the other hand, it is important to comment that, in the case of systems with low ionization degree, most of the data provided by the instrument reported that it was necessary to review the measurement quality report. It was found that the correlation function did not always have a good fit and that at the end of its baseline, atypical signals appeared. Such information suggests that those particles that could not be measured by the instrument are, in fact, large particles that tend to aggregation over time [37,38,39]. On the contrary, the systems with medium and high ionization degrees could obtain appropriate measurements with good adjustment to the correlograms. However, in cases where the particle size increased over time, those distortions of the correlogram baseline were also observed, indicating aggregation effects.

Therefore, all these particle size and polydispersity results obtained by DLS are very interesting and relate very well to those previously observed in turbidimetric solubility, as well as zeta potential, pH, and electrical conductivity. In this way, all the results describe that, in fact, there is a high polyelectrolytic dynamism, with a marked effect between the polymeric ionization degree and inter-polymer aggregation. Accordingly, systems with a low ionization degree tend to be poorly solvated by the aqueous medium, generating multiple systems corresponding to heterogeneous suspensions (coarse colloids). Such conditions lead to interparticle aggregation, forming flocs or clots in systems with low values of zeta potential, as described in the DLVO theory [40,41]. In relation to time, this was shown not to affect the thermodynamic equilibria formed between the polyelectrolyte and the aqueous medium, leading to the pH remaining almost unchanged at ~6. This result can be explained considering that there is a minimum amount of solubilized polyelectrolyte chains and therefore, the amount of available carboxylate groups to form such thermodynamic equilibria is very limited. 

**Figure 5:**
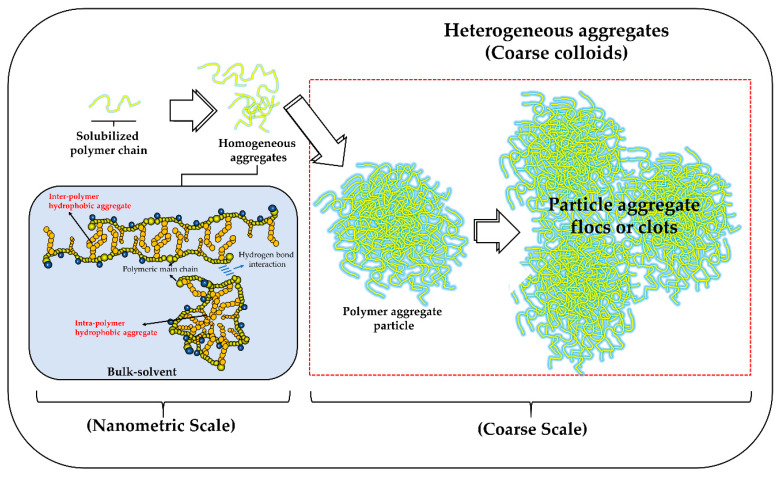
Scheme of formation of inter-polymeric aggregates at different size scales.

In contrast, polyelectrolyte systems with medium and high ionization degrees, showed that these can be between two states of interaction with the aqueous medium (solvation and wetting) [42,43] at the same time, forming different types of particles. In the case of systems with particle sizes on a nanometric scale, the polyelectrolytes are highly solvated, generating a homogeneous colloidal condition (polymer solution), while with coarse particle sizes, they are as wet-solid–liquid suspensions. In this way, the increase in the ionization degree leads to an increase in the zeta potential, avoiding the inter-polymer aggregation and decreasing the particle size. While systems with large sizes form colloidal suspensions that can be wetted and that depending on the zeta potential, can reach a condition of stationary stability or inter-polymer aggregation. Consequently, polyelectrolyte systems with high ID show that regardless of time, they always describe a polymer solution type behavior, where the polymer chains are strongly solvated and where their aggregation is mediated by the interaction of the lateral alkyl chains, similarly as it happens in the aggregation of surfactants (Figure 5). Finally, in order to provide further arguments regarding such aggregation phenomena, the change in surface tension regarding the polyelectrolyte concentration was evaluated, as described below.

### 3.3. Surface Tension Measurements

**Figure 6:**
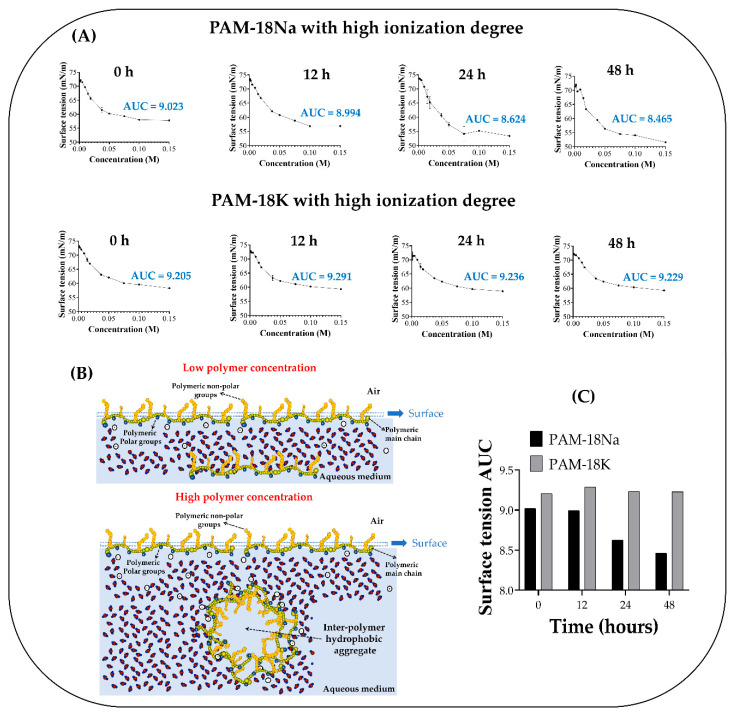
(**A**) Change in surface tension regarding to the concentration of PAM-18Na and PAM-18K polyelectrolytes with high ionization degree (ID) in aqueous media. (**B**) Scheme of polyelectrolyte located in the superficial zone. **C**) AUC values of the surface tension vs. polymeric concentration in different times.

First, the effect of polyelectrolytes with low and medium ionization degrees on surface tension was evaluated. However, no convincing change was observed regarding to the surface tension value of pure water (~72 dynes/cm). This result can be explained considering that as the concentrations where the homogeneous systems are formed is so low, it is very hard to appreciate a change in the surface tension. On the contrary, the polyelectrolytes with a high ionization degree allowed preparing a set of solutions with concentrations between 0.01 and 0.15 M and where it was possible to observe a considerable change in surface tension, as shown in Figure 6A. For both polyelectrolytes, the results show the typical behavior of a surfactant system, which is consistent, considering that the PAM-18Na and PAM-18K polyelectrolytes with high ionization degree are indeed, amphiphilic structures, where the two acid carboxylic and carboxylate groups form the polar region, while the hydrocarbon alkyl chain forms the non-polar region [21]. 

As a result, to understand this phenomenon of aggregation over time, it is necessary to consider the different types of conformation that these can achieve in an aqueous medium. Thus, when the polyelectrolytes have a high ionization degree due to their carboxylate groups, these lead to an extension of the polymeric main chain in order to decrease the electrostatic repulsion, avoiding the folding between their own alkyl chains (intra-polymeric aggregation) (Figure 5). These extended conformations can also drive other types of processes similar to the micellar aggregation given by surfactant molecules [44,45,46,47].

In this way, at very low concentrations, the polymer chains are widely separated from each other, allowing them to achieve high mobility and the ability to migrate to the surface area, where they can accommodate reaching a highly favored thermodynamic conformation [48]. This way, on the air–water surface, these amphiphilic polymers can locate their alkyl chains towards the air (non-polar character) and their carboxylic and carboxylate acid groups towards the aqueous medium (polar character) until it is completely saturated (Figure 6B). Subsequently, the polyelectrolyte migrates into the solution, forming a dynamic state of migration between both places in the system. Then, the increase in the polymer concentration means that they can only be located within the solution, increasing the number of alkyl side chains and, therefore, the hydrophobic effect. Taking this into consideration, in order to avoid such an effect, these amphiphilic polymers begin to aggregate, adopting new conformational states that lead to a more favorable thermodynamic condition. 

Thus, the hydrophobic effect is replaced by a new configurational state, which is formed by several attractive binding forces, such as van der Waals interactions given between the alkyl side chains and the ion-dipole and hydrogen bonds interactions given between the carboxylic acid/carboxylate groups and the solvent. In order to demonstrate whether these polyelectrolyte systems can change over time, the values of the areas under the curve of the surface tension profiles regarding to the polymer concentration were determined (Figure 6C). The results show that in the case of the PAM-18K polyelectrolytes, the AUC values tend to be very similar to each other, while with the PAM-18Na polyelectrolyte, this value decreases over time, indicating a slight loss of surfactant capacity. This result is very consistent, considering the previous values of zeta potential, pH, and electrical conductivity, which showed that PAM-18Na tends to generate less polarized interfaces than PAM-18K and where counter ions are more strongly united in double electrical layer. In this way, the PAM-18Na polyelectrolyte tends to form with their counterions, structures separated by intimate ion pair and solvent-separated ion pair, which can strongly shield the charge of the polyelectrolyte, decreasing its hydrophilicity and therefore its amphipathicity.

Lastly, it is important to mention that the statistical analysis indicated several significant differences, depending on the physicochemical property of the polyelectrolyte in relation to the polymeric ionization degree and the time. In the case of pH, electrical conductivity and AUC values, these displayed significant differences regarding to the ID and the time. In contrast, particle size and zeta potential showed significant differences only in some cases. Particle size displayed significant differences only in polymers with high ID, whilst the zeta potential described significant differences in polymers with low and high ID. The summary of the statistical analysis results (Tables S1–S5) is presented in the Supplementary Materials.

## 4. Conclusions

The increase in the polymeric ionization degree leads to an increase in the polyelectrolyte apparent solubility, being greater in those polyelectrolytes that contain the potassium counterion. Furthermore, the solvation effect generated between the polymeric systems and the aqueous medium depends on a balance between attractive ion-dipole and hydrogen bond interactions and the hydrophobic effect. Thus, in systems with a high ionization degree, attractive interactions prevail and the polymer dissolves, forming homogeneous systems with smaller particle sizes and polydispersity. On the contrary, in the polymeric systems with a low degree of ionization, the hydrophobic effect prevails, and the polyelectrolytes are wetted, forming coarse colloidal aggregates (flocs and clots) that are very polydispersed with large particle sizes. Likewise, it was found that polyelectrolytes with high ionization degrees may form different types of aggregates in aqueous solution, which depend on the type of polyelectrolyte, as well as time, describing a process like micellization. Also, it was found that these polyelectrolytes change their initial thermodynamic equilibria, where the polyelectrolytes that acted as strong (high ID) and relatively strong (medium ID) are transformed into weak polyelectrolytes (low ID) over time. Finally, the PAM-18Na and PAM-18K polyelectrolytes described to be extremely dynamic systems that lead to different aggregation processes in solution (homogeneously), as well as in dispersion (heterogeneously). Therefore, these results show that, in the case of amphiphilic alternating polymeric materials, such as the salts of poly (maleic acid-alt-octadecene), time is a critical variable that must be considered and controlled.

## Supplementary Materials

The following are available online at https://www.mdpi.com/2073-4360/12/5/1036/s1, Table S1: Statistical analysis (One Way ANOVA)—Conductivity (µS/cm); Table S2: Statistical analysis (One Way ANOVA)—pH; Table S3: Statistical analysis (One Way ANOVA)—Zeta Potential (mV); Table S4: Statistical analysis (One Way ANOVA)—Size (nm); Table S5: Statistical analysis (One Way ANOVA)—AUC of surface tension vs polymer concentration.
