# Supplementary material for: Relationship between the Ionization Degree and the Inter-Polymeric Aggregation of the Poly(maleic acid-alt-octadecene) Salts Regarding Time
Source: Polymers (Basel). 2020 May 2;12(5):1036. doi: 10.3390/polym12051036 (PMC7284458; doi:10.3390/polym12051036)
Supplement: Supplementary file 1 [file polymers-12-01036-s001.pdf]

Relationship between the ionization degree and the inter-polymeric aggregation of the poly(maleic acid-*alt*-octadecene) salts regarding time.

Support material

Table S1: Statistical analysis (One Way ANOVA) - Conductivity (µS/cm)

ID	Time (h)	P-Value	Significant difference
Low	0	0.000	Yes
	12	0.000	Yes
	24	0.000	Yes
	48	0.628	No
Medium	0	0.321	No
	12	0.000	Yes
	24	0.062	No
	48	0.000	Yes
High	0	0.000	Yes
	12	0.000	Yes
	24	0.000	Yes
	48	0.000	Yes

Table S2: Statistical analysis (One Way ANOVA) – pH

ID	Time (h)	P-Value	Significant difference
Low	0	0.017	Yes
	12	0.016	Yes
	24	0.006	Yes
	48	0.007	Yes
Medium	0	0.093	No
	12	0.000	Yes
	24	0.009	Yes
	48*	0.000	Yes
High	0	0.020	Yes
	12	0.000	Yes
	24	0.000	Yes
	48	0.000	Yes

Table S3: Statistical analysis (One Way ANOVA) - Zeta Potential (mV)

ID	Time (h)	P-Value	Significant difference
Low	0	0.000	Yes
	12	0.016	Yes
	24	0.022	Yes
	48	0.269	No
Medium	0	0.180	No
	12	0.451	No
	24	0.015	Yes
	48	0.773	No
High	0*	0.118	No
	12	0.448	No
	24	0.811	No
	48	0.289	No

Table S4: Statistical analysis (One Way ANOVA) - Size (nm)

ID	Population	Time (h)	P-Value	Significant difference
Low	1	0	0.029	Yes
		12	0.717	No
		24	0.000	Yes
		48	0.002	Yes
Medium	1	0	0.124	No
		12	0.015	Yes
		24	0.205	No
		48	0.000	Yes
	2	0	0.316	No
		12	0.701	No
		24	0.559	No
		48	0.004	Yes
High	1	0	0.023	Yes
		12	0.267	No
		24	0.001	Yes
		48	0.001	Yes
	2	0	0.008	Yes
		12	0.006	Yes
		24	0.007	Yes
		48	0.535	No

Table S5: Statistical analysis (One Way ANOVA) -AUC of surface tension vs polymer concentration

**ID**	**Time (h)**	**P-Value**	**Significant difference**
High	0	0.003	Yes
	12	0.000	Yes
	24	0.001	Yes
	48	0.000	Yes
